# Symptom reduction and patient satisfaction after surgical therapy of Meralgia paresthetica – A bicentric retrospective analysis^☆^

**DOI:** 10.1016/j.bas.2026.106060

**Published:** 2026-04-18

**Authors:** Lena Minzenmay, Eva-Maria Boom, Andrej Pala, Gregor Antoniadis, Maria-Teresa Pedro, Ute Marlies Bäzner, Andreas Knoll, Christian Rainer Wirtz, Jürgen Beck, Christoph Scholz, Marc Hohenhaus

**Affiliations:** a Department of Neurosurgery, University of Ulm, BKH Günzburg, Günzburg, Germany; b Department of Neurosurgery, Medical Center - University of Freiburg, Faculty of Medicine, University of Freiburg, Freiburg, Germany

**Keywords:** Meralgia paresthetica, Nerve compression syndrome, Neurolysis, Decompression, Neurectomy, Lateral femoral cutaneous nerve

## Abstract

**Introduction:**

Surgical treatment for Meralgia paresthetica is indicated in patients showing deficient effects of conservative interventions and merely temporary positive responses after local infiltrations of the lateral femoral cutaneous nerve (LFCN).

**Research question:**

To improve clinical outcome data, especially with focus on the long-term effect of such procedures, we evaluated patients in two neurosurgical centers with regard to their satisfaction and reduction of symptoms.

**Methods:**

Retrospective analysis of perioperative characteristics and follow-up telephone survey concerning subjective satisfaction after surgery using the Patient Satisfaction Index (PSI), subjective complaint reduction (percentage) and pain response (Numeric Rating Scale). In addition, health-related quality of life and sensation at the thigh were evaluated. Regression analysis concerning risk factors for an insufficient outcome was applied.

**Results:**

A total of 108 surgeries were performed on 94 patients (50% female, median age 56 years, BMI 27.9 kg/m^2^). 98 operations were neurolysis of the LFCN, 4 secondary neurectomies and 6 other revisions. A satisfying outcome at follow-up (PSI 1 + 2) was achieved at 84.4% of the patients, whereas 10.4% had no relevant symptom reduction (PSI 4). The average permanent complaint reduction was 72.7%. Concerning the residual pain intensity at the thigh at rest/under strain, 80.6%/72.0% showed favorable values (NRS≤3). Regression analysis revealed nicotine abuse as independent risk factor for an unsatisfactorily outcome (OR 6.490; p = 0.027)

**Conclusions:**

Surgical treatment of Meralgia paresthetica significantly improves associated complaints in the majority of patients leading to a high amount of satisfying results. Nicotine abuse seems to be a risk factor for insufficient outcomes.

## Highlights

• 84.4% of surgically treated Meralgia paresthetica patients were satisfied.
• The average permanent complaint reduction was 72.7%.
• About 80% of the treated patients showed a residual NRS≤3 after surgery.
• Only 10.4% had no relevant symptom reduction.
• Nicotine abuse was an independent risk factor for an unsatisfactorily outcome.

## Abbreviations

BMI: Body Mass Index
CI: Confidence Interval
IQR: Interquartile Range
LFCN: Lateral Femoral Cutaneous Nerve
MP: Meralgia paresthetica
NRS: Numeric Rating Scale
OR: Odds Ratio
PSI: Patient Satisfaction Index
QoL: Quality of Life
SF-8: Short Form Health Survey 8

## 1 Introduction

Meralgia paresthetica (MP) is a rare compression syndrome of the purely sensory lateral femoral cutaneous nerve (LFCN), whereas reliable incidences are not available, so that this diagnosis might be underrepresented. It was first mentioned 1895 and associated to their first describers also known as Bernhard-Roth syndrome (Bernhardt, 1895; Roth, 1895). MP is diagnosed by a typical clinical presentation with pain, sensory loss and/or paresthesia at the anterolateral thigh under exclusion of a spinal symptom origin (Parisi et al., 2011; Scholz et al., 2023). The incidence is reported with up to 43/100,000 patient years, whereas an increasing incidence is expected due to the association with obesity and co-morbidities like diabetes, that are even more frequent due to the popular burden of metabolic syndrome (Flegal et al., 2010; Parisi et al., 2011; van Slobbe et al., 2004). Usually, there is an entrapment of the LFCN under the inguinal ligament and/or the fascia of the abdominal wall and upper thigh, mostly direct laterally of the anterior superior iliac spine, whereby there are some anatomic variants in the course of the nerve (Carai et al., 2009). Less frequently, lesions of the LFCN may occur post-traumatic or iatrogenic, e.g. after hip arthroplasty (de Ruiter et al., 2023). The associated neuropathic pain syndrome can become chronic and is frequently neither adequately diagnosed nor treated. Treatment modalities include the reduction of risk factors like obesity or tight clothing, manual or physical therapy, WHO°I analgesics as well as local injections (Scholz et al., 2023). Surgery is recommended if conservative management fails and primarily retrospective data could show sufficient pain reduction through these interventions.

The two main surgical procedures are a decompression of the LFCN with neurolysis and resection of constricting tissue on the one hand and the primarily destructive neurectomy of the LFCN on the other hand (de Ruiter and Kloet, 2015; Malessy et al., 2019). These two established techniques exist in parallel, with the type of treatment for each individual patient depending primarily on the surgeons' choice (Scholz et al., 2023). Data quality concerning the operative success rates within the literature is poor and inconsistent, based on mostly retrospective case series or small observational studies (Khalil et al., 2008, 2012; Lu et al., 2021). There seems to be a tendency for a better pain reduction when the nerve is dissected, whereas the sensory function at the lateral thigh is therefore irretrievably lost (Khalil et al., 2008, 2012; Lu et al., 2021). This can be saved in parts of the patients treated by neurolysis as a non-destructive procedure, with the potential disadvantage for symptom recurrence and necessary reoperation. Data on patients’ subjective satisfaction after such an intervention are completely lacking. The superiority or equality concerning both surgical procedures remain unclear as well, leading to uncertainty in treatment recommendation of affected patients (Dengler, 2023; Payne et al., 2017).

In order to improve the quality of data concerning the long-term outcomes after surgical treatment of MP, we decided to conduct a retrospective evaluation of a larger patient cohort, including two centers with a higher treatment frequency. This should also provide a basis for preparing a comparison of both surgical procedures in a multicenter design in the future. So, the aim of our study in particular was to evaluate the symptom reduction and patient satisfaction in surgically treated MP patients.

## 2 Materials and methods

**Fig. 1:**
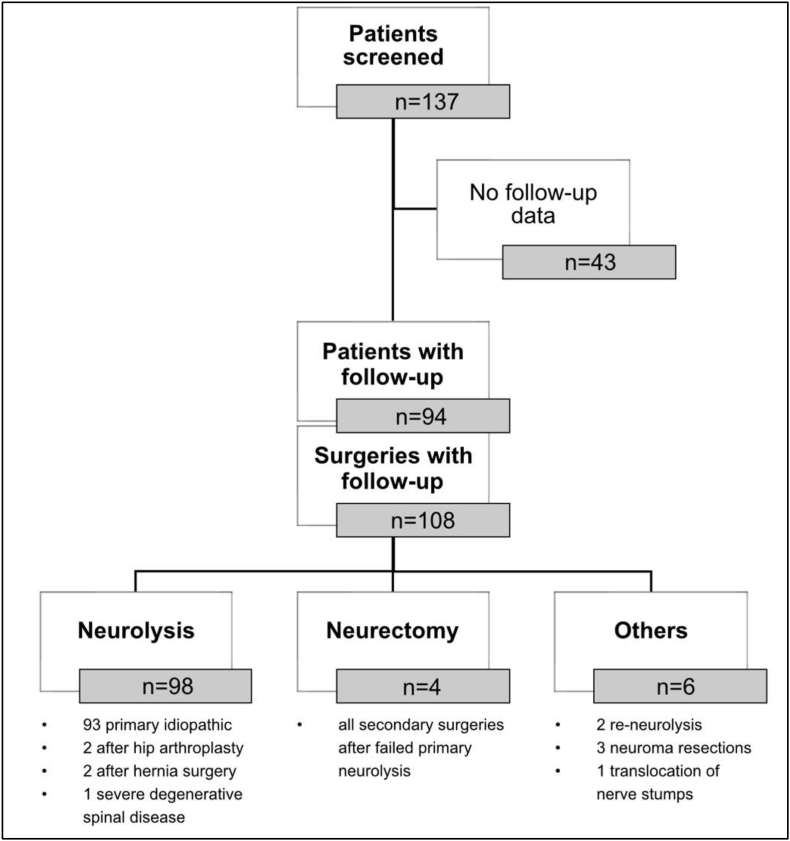
Overview of all patients that received surgery on the LFCN at both centers, available follow-up data and the types of procedures that were performed on the evaluated patients.

This is a pooled, retrospective evaluation of two higher treatment frequency university hospitals including all patients who underwent surgical treatment of MP between 2010 and 2023. The study was approved by the Ethical Review Committee of the University of Ulm, Germany (85/24). Overall, 137 patients were treated surgically in both centers within the mentioned time period, whereas 94 patients could be analyzed due to available follow-up data (Fig. 1).

### 2.1 Baseline data

Clinical data were recorded from the medical reports (age, gender, surgical procedure with side and date, primary diagnosis and prior surgery) and premedication forms (Body Mass Index (BMI), secondary diagnoses, medications). Duration of surgery was extracted from local surgical documentation.

### 2.2 Surgical treatment

All patients received a probatory infiltration of the LFCN with local anesthetics. In case of a positive response to the infiltration, even for a short period of time, the operation was performed. Like mentioned above, two established surgical treatments exist for MP, whereas the most common primary approach is the neurolysis of the LFCN within the most European countries, so that this was the primary procedure at our departments as well. For decompression and neurolysis of the LFCN, supra- and infrainguinal approaches were performed. Neurectomy was only performed secondarily in cases of insufficient symptom reduction due to the first procedure or atypical MP with an already destroyed nerve.

### 2.3 Standardized follow-up survey

After identification of all patients receiving surgical MP treatment, a standardized questionnaire (Supplement 1) and associated telephone survey concerning the long-term outcome was conducted in 2024 and 2025. The patients were asked for their constant or temporarily effect through the surgery as well as the percentage of complaint reduction. The presence of current pain at the groin and thigh at rest and under strain were queried by the Numeric Rating Scale (NRS). Additionally, the intake of painkillers was scanned. In addition, patients were asked about their sensory function at the ventrolateral thigh, whether it had changed, whether hypesthesia persisted, and whether this is bothersome or not. The survey contained questions about weight progression, the occurrence of wound healing disturbances, secondary nerve compression syndromes and even concerning MP symptoms at the other side. Patient satisfaction and health-related quality of life (QoL) were addressed by the four-item Patient Satisfactory Index (PSI, see Supplement 1) and Short Form Health Survey 8 (SF-8). Patients in one treating center also completed the EQ-5D-5L questionnaire (Graf et al., 1998).

### 2.4 Outcome categorization

Based on the assessed PSI, the percentage of complaint reduction as well as the residual NRS values, we categorized the outcomes arbitrarily as follows:

PSI values of 1 and 2 were defined as “satisfactorily” outcome, whereas these patients would undergo surgery for the same effect again. PSI 4 was defined as “unsatisfactorily” because these patients showed no effect through the surgery. We clustered the patients concerning their reduction of complaints through the surgery in “sufficient” meaning a treatment effect of more than 50%, “acceptable” with an effect of 20-50% and “insufficient” for less than 20% symptom reduction. At least, the residual pain at follow-up was categorized as “favorable” outcome in NRS≤3 as low pain intensity for patients with often chronic pain, “intermediate” for NRS values of 4 or 5, and “unfavorable” with higher pain intensities of NRS>5.

### 2.5 Statistical analysis

Statistical analysis was performed using SPSS Statistics 29.0 (IBM Corporation, Armonk, New York, USA) and Microsoft Excel version 16 (Microsoft Corporation, Redmond, Washington, USA). For the SF-8 questionnaire, the Physical Component Summary (PCS) and Mental Component Summary (MCS) were calculated. EQ-5D-5L health-related QoL scores were processed using R (4.2.3). For each patient an EQ index was calculated using eq (5d) R package and the German value set (Ludwig et al., 2018; Morton and Nijjar, 2025). Normal distribution was assessed by Shapiro-Wilk-test and due to predominantly non-normal distributed values, Median and Interquartile Range (IQR) was stated. Sankey plot was built with SankeyMATIC.com and modified with Affinity Designer (Serif Europe Ltd., Nottingham, United Kingdom). Risk factor analyses for an unfavorable outcome was done by binominal logistic regression.

## 3 Results

For data analysis we could include 94 patients which overall received 108 surgeries on the LFCN, predominantly as first-time neurolysis (90.7%, Fig. 1). The other ten surgical interventions were neurectomies (n = 4, 3.7%), re-neurolysis (n = 2, 1.9%), resections of stump neuromas (n = 3, 2.8%) and one translocation of nerve stumps to deeper layers (n = 1, 0.9%).

Concerning patient stratification, we have to address the idiopathic versus non-idiopathic causes for the MP and in the second stage the first-time and revision surgeries with the LFCN as target. In 99 of the 108 operations (91.7%), it was the first surgical intervention on the LFCN. This results in nine revision procedures, matching to the above mentioned “non-primary neurolysis cases”. The one discrepant case was an iatrogenic MP patient after spongiosa extraction at the iliac crest and resulting primary neurectomy because of an intraoperative destroyed nerve. In addition to this case, there were five other non-idiopathic MP patients within the cohort, caused by the following reasons: two iatrogenic lesions through hip arthroplasty, two iatrogenic lesions after inguinal hernia surgery and at least one attempt to reduce chronic pain based on a degenerative disease of the lumbar spine. All other surgeries were based on idiopathic MP without prior surgery on the LFCN (n = 93, 86.1%). Additionally, we have to provide, that five patients were treated concerning MP on both sides. Two patients of them received revisions on one side (one neurectomy and one re-neurolysis), whereas three patients were treated on both sides without revision surgery. An overview concerning the patient distribution on the different procedures is given in Fig. 1.

Baseline parameters concerning the surgical interventions showed a median age at the time of surgery of 56 years (IQR 14, range 22 – 84 years) and gender distribution was exactly equal (50%). The median BMI was 27.9 kg/m^2^ (IQR 7.1, range 18.6 – 46.3 kg/m^2^). Hypertension was present in 54 cases (50%), whereas diabetes (n = 10, 9.3%) and nicotine abuse (n = 17, 25.9%) were less frequent. Anticoagulants were given in 17 patients (15.7%), whereas patients paused the medication for surgery if possible. Seven patients (6.5%) showed an additionally carpal tunnel syndrome, but no other nerve compression syndromes. The was a predominance concerning the right side treated (54.6% versus 45.4%) and the median duration of surgery was 50 min (IQR 20, range 21 – 165 min). Seven (6.5%) of all patients showed a wound healing disturbance, treated by antibiotics without the need for surgical revision. The median time from the last surgical intervention to follow-up was 37.5 months (IQR 89, range 1 – 164 months). Patients reported constant body weight in 37%, whereas most patients showed an increase (45.2%, range 1 – 28 kg) and only 11.8% could reduce their weight (range 2 – 10 kg).

**Fig. 2:**
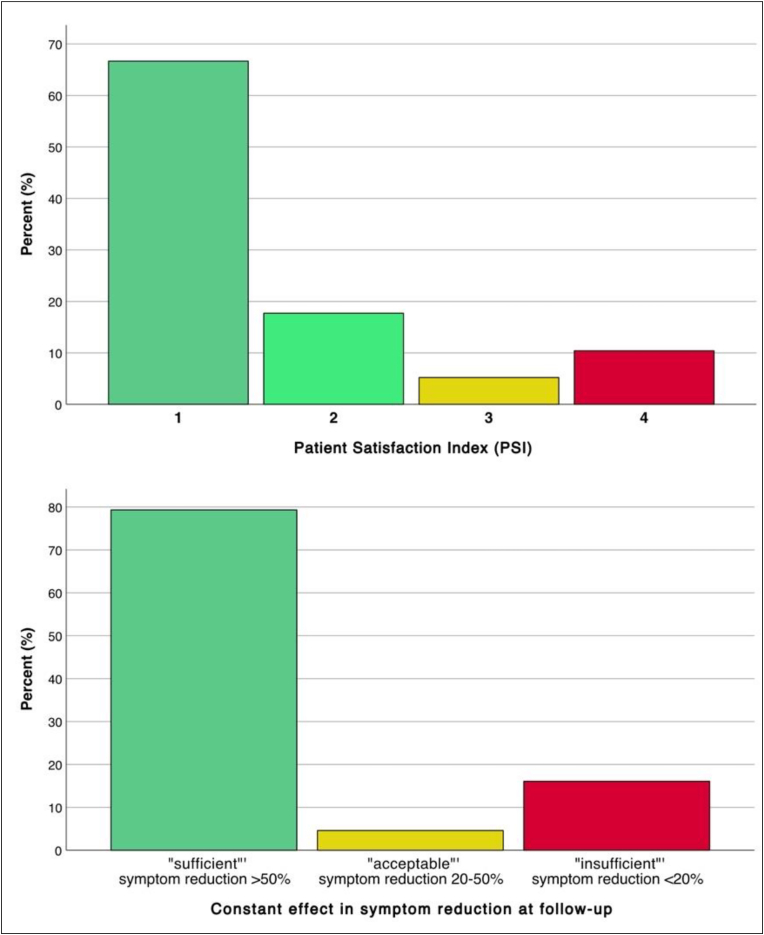
Subjective clinical outcome of all patients after surgical treatment of Meralgia paresthetica via Patient Satisfaction Index (PSI, above) and percentage in symptom reduction (below). Even in cases where the outcome after surgery was unsatisfactory/insufficient, none of the patients experienced a worsening of symptoms.

The overall subjective patient satisfaction after surgically treated MP is shown in Fig. 2. Therefore, 84.4% of the patients were satisfied with the treatment (PSI 1 + 2). There were 10.4% of the patients that had unsatisfactorily no relevant symptom reduction (PSI 4). The average percentage of symptom reduction at follow-up was at 72.7%. When applying the outcome categorization for the extent of symptom reduction, the treatment could achieve 79.3% “sufficient”, 4.6% “acceptable” and 16.1% “insufficient” results (Fig. 2). In 20 patients this value was missing.

**Fig. 3:**
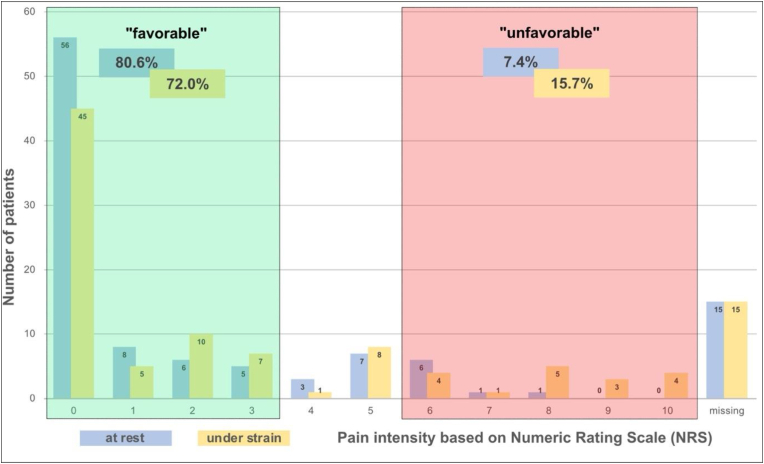
Bar chart on the residual pain intensity (NRS) at the thigh at the follow-up survey after surgical treatment of Meralgia paresthetica. The favorable outcomes are marked in slight green and the unfavorable outcomes in slight red. Even in cases where the outcome after surgery was unfavorable, none of the patients experienced a worsening of symptoms.

As a kind of more objective parameter, the residual pain intensity was specified by NRS at rest and under strain. The results are shown in Fig. 3. Based on the outcome categorization, 80.6% showed a “favorable” outcome at rest and 72.0% under strain. Of those patients, 56 (60.2%, at rest) as well as 45 (48.4%, under strain) patients could be declared as “pain-free” (NRS 0).

Beside this, there were also patients that reported residual pain at the groin (30.1% at rest and 36.6% under strain), whereas the median pain intensity was low (NRS 2 (IQR 2) at rest and NRS 3 (IQR 4) under strain). Respectively, within the most patients, local pain already disappeared (69.9% at rest and 63.4% under strain).

**Fig. 4:**
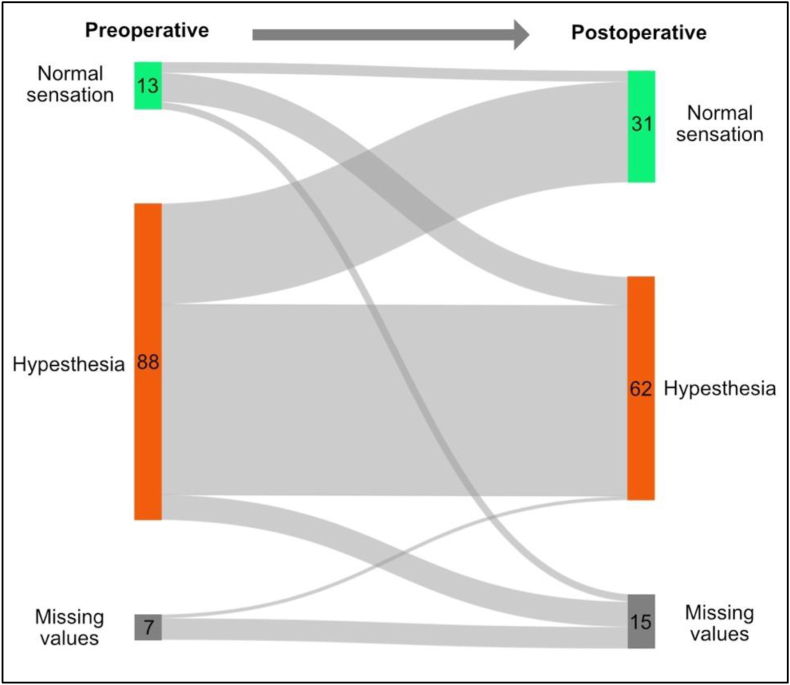
Sankey plot concerning the development of the sensory function at the ventrolateral thigh.

Concerning the sensation at the ventrolateral thigh, 88 patients (81.5%) showed already hypesthesia prior to surgery, whereas 13 patients (12.0%) showed a normal sensation at the thigh. In seven patients the preoperative information was missing. Postoperatively, 28 patients showed a disappeared hypesthesia, whereas eight patients showed a new sensory reduction at the thigh. The distribution from pre-to postoperative is shown in Fig. 4. Of all 62 patients that showed a postoperative hypesthesia, only 27 (43.5%) found the reduction in sensation bothersome. All the patients that bothered from the hypesthesia were treated with neurolysis, so that no patient receiving neurectomy or neuroma resection suffered from the sensory loss. Only two patients with a bothersome hypesthesia showed preoperatively no sensory reduction, in all other cases the hypesthesia was already present prior to surgery.

**Fig. 5:**
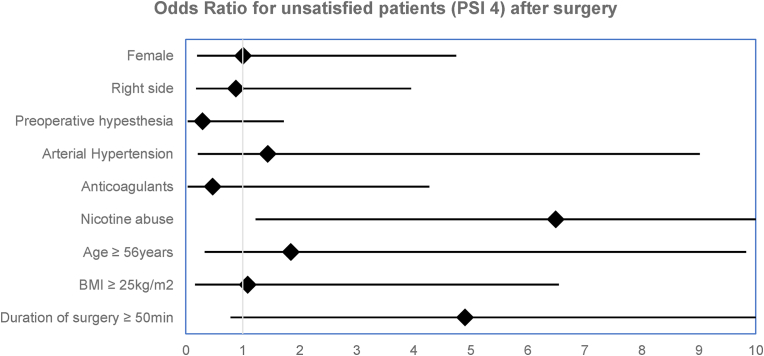
Forrest plot showing the Odds Ratio (95-CI) for the risk for an unsatisfactorily outcome (PSI 4) after surgical Meralgia paresthetica treatment for the nine factors entered into the regression model. The co-morbidities diabetes mellitus and other nerve compression syndromes had to be excluded because of the limited incidence within our study population.

We screened for risk factors for an unsatisfactorily outcome (PSI 4) through binary logistic regression and found nicotine abuse (OR 6.490; 95-CI 1.242 – 33.916; p = 0.027) to be significantly, and a duration of surgery longer than 50 min (OR 4.902; 95-CI 0.802 – 29.959; p = 0.085) to be associated as a trend. All analyzed factors are shown within Fig. 5.

Using the percentage of treatment effect for the risk factor screening, there were no significant associations. Based on the residual pain (NRS>5 as “unfavorable” outcome), we could detect a trend for female patients to tend for an unfavorable outcome for their pain at the thigh under strain (OR 2.513, 95-CI 0.900 – 7.015, p = 0.079). All other mentioned factors as well as the residual pain intensity at rest showed no significant results.

**Fig. 6:**
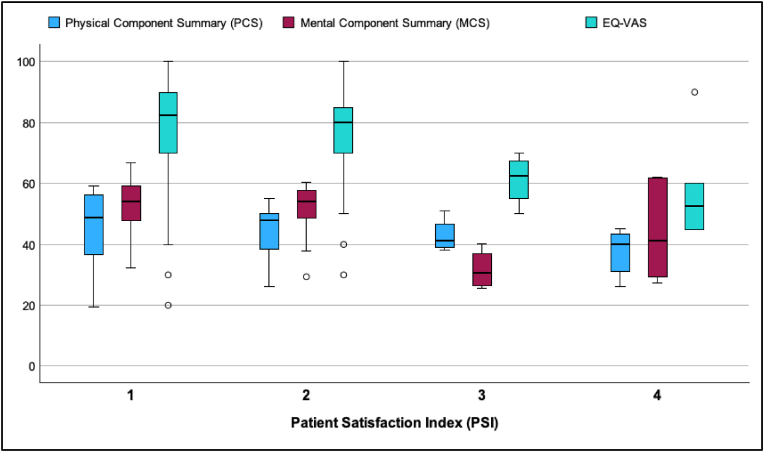
Clustered boxplots for Physical Component Summary (PCS) and Mental Component Summary (MCS) values as well as the EQ-VAS split for the different patient satisfaction classes of the PSI. Outliers (1.5 times interquartile range) are shown as circles.

Concerning the postoperative QoL measured with the SF-8, the median value for PCS postoperatively was 45.64 (IQR 15.58; range 19.44 – 59.31; n = 91) and MCS was 52.49 (IQR 14.69; range 25.51 – 66.91; n = 91). Splitting PCS and MCS measures concerning the patient satisfaction (PSI) showed decreasing values for unsatisfied patients (Fig. 6). Complete EQ-5D-5L questionnaires were available for 70 patients after surgery, with a median EQ index for the whole study population of 0.917 (IQR 0.147, range 0.108 – 1.000) and EQ-VAS rating of 80.0 (IQR 36; range 5 – 100). Splitting these ratings concerning the treatment satisfaction showed decreasing values if the patients were not satisfied, as expected (see Fig. 6 for EQ-VAS). These correlations reflect a significant increase of the QoL when patients were satisfied with the surgery.

## 4 Discussion

Our retrospective evaluation of 108 surgeries for Meralgia paresthetica could reveal efficient treatment results with 84.4% satisfied and up to 80.6% patients with a low residual pain intensity of NRS≤3 at follow-up. These are very good results for patients who often suffer from chronic pain that affects their everyday lives. We found nicotine abuse to be associated with a worse clinical outcome, which represents an influential factor for affected patients.

Basically, our study population reflects a classical MP cohort with tendentially pre-obese or obese patients (BMI 27.9 kg/m^2^; IQR 7.1; range 18.6 – 46.3 kg/m^2^), which is a known risk factor for MP, and in average middle-aged adults (56 years; IQR 14). The most recent meta-analysis of Lu et al. showed a median patient age of 49 years (range 11 – 70 years) (Lu et al., 2021). The mean BMI in another retrospective trial on 167 MP patients was 27 kg/m^2^ (range 17 – 46 kg/m^2^) (Benezis et al., 2007). Beside patients age and obesity, diabetes mellitus is another known risk factor for MP, whereas our cohort showed only 9.3% compared to other studies with up to 28% (Parisi et al., 2011).

There are three goals for the long-term outcome of surgical MP treatment: pain reduction, sensory restoration, and QoL improvement. To summarize based on the pre-existing literature, pain reduction seems to be most effective in neurectomy, whereas a sensation restoration is not possible. Data on QoL and activities of daily living are scarce (Reuter et al., 2025).

Within the most European countries, neurolysis is the predominant surgical intervention in MP, consistent with our patient cohort (Schönberg et al., 2023). This bases at the non-destructive concept, whereas precise data on treatment efficacy are missing for the different surgical strategies and therefore guideline recommendations. Within the United States the neurectomy seems to be more popular because of a higher reported success rate, reduced need for secondary surgeries and therefore cost savings for affected patients. But, also the available literature within the United States shows a clear tendency towards neurolysis at the first step, when MP has to be treated surgically. The meta-analysis of Lu et al., displaying overall 670 MP patients with interventional treatments, reported a five-time higher rate of neurolysis compared to neurectomy (496 versus 96 patients) (Lu et al., 2021). There was unfortunately no statement if the neurectomies were revision procedures or primary treatments. In our cohort, only four secondary neurectomies and one re-neurolysis on respectively five patients were done because of no sufficient symptom reduction after primary neurolysis.

The evidence concerning the treatment effect of the different interventional techniques is low and data quality poor (Payne et al., 2017; Scholz et al., 2023). A major restriction concerning prior trials and case series are the inconsistent definitions of treatment success. A major complaint to address is the pain at the ventrolateral thigh, whereas specific pain outcomes for MP are not established yet (Lu et al., 2021). There are trials reporting “complete or partial pain relief” or “decreased symptoms” as good outcomes. Our trial is the first that determines the treatment results in a highly standardized manner. Subjective success was defined by PSI in a Lickert scale and the extent of symptom reduction as percentage. Additionally, the residual pain is documented by NRS as a highly comparable value. In our opinion, patient-related outcome measures like PSI or health-related QoL scores, have to be combined with objective scales, like NRS, to classify the outcome of surgically treated patients as precise as possible, especially in chronic pain diseases.

Within our study population, 84.4% of the patients were satisfied with the treatment based on PSI (1 + 2). Another 5.2% had some kind of symptom reduction, but would not undergo surgery for the same result (PSI 3). Clustering the patients’ outcome concerning their reduction of complaints, 83.9% showed sufficient or acceptable results, which also reflects a very efficient treatment in this partially chronic pain patients. The meta-analysis of Lu et al. stated 63% (CI 56-71%) of patients after decompression as “pain-free”, which is congruent with our results (Lu et al., 2021). Nevertheless, even if the patients are not completely “pain-free”, the surgery can reveal satisfying results. In our evaluation, over 80% of the patients were satisfied, whereas “only” about 60% were absolutely pain-free. We would state an NRS≤3 as treatment goal for the pain at the thigh for surgically treated MP patients.

There was no prior study that distinguished between residual pain intensity at rest and under strain, whereas this seems to be important for the all-day life of the patients. Based on the NRS, 92.6% showed a “favorable” (80.6%) or at least “intermediate” (12.0%) outcome at rest and 84.3% a “favorable” (72.0%) or at least “intermediate” (12.3%) outcome under strain. A recent evaluation of Reuter et al. of 26 patients also receiving neurolysis of the LFCN showed also a very effective treatment with a mean pain intensity reduction from NRS 6.6 (±1.9) to 0.7 (±1.1) (Reuter et al., 2025). 76.9% of patients achieved complete pain elimination. Overall, 88.5% stated their outcome as good or even excellent.

Within the literature, an unsatisfied patient proportion of about 20% is described. Lu et al. stated one out of five MP patients without complete pain relief and Benezis et al. reported 22% patients without improvement of symptoms (Benezis et al., 2007; Lu et al., 2021). We could declare an overall matching number of not satisfactorily results within our retrospective cohort, whereas only 10.4% of the patients remained without any relevant symptom reduction. One mentioned reason for treatment failures within the literature is the possible formation of stump neuromas, whereas only three neuroma resections occurred in our cohort, two of them within one patient and the other patient after an iatrogenic lesion of the nerve and prior neurolysis. Another cause could be the anatomical variation of the LFCN with atypical courses or multiple branches, so that the nerve was not completely treated through the surgery or even were not exactly found (Carai et al., 2009; Keegan and Holyoke, 1962). It was already stated, that patients with MP would show a higher rate of atypical courses of the LFCN, which might lead to an increased rate of misidentifications during surgery (Johnson et al., 2025). Within the study of Benezis et al. an atypical course of 17% is reported (Benezis et al., 2007).

In our opinion, differential diagnosis and co-morbidities play another substantial role for treatment success. There might be a residual risk for treating overlapping entities, like hip pathologies or degenerative spinal diseases. Additionally, these mostly degenerative diseases can occur simultaneously and therefore reduce the satisfaction rates of treated patients, even if the MP treatment reduces the thigh affection. In our cohort, we had a patient with a severe spinal degeneration and multiple prior spinal surgeries, that were treated for MP as attempt to reduce his pain at the thigh without success (PSI 4). Another patient showed delayed symptom reduction only after surgical treatment of an inguinal hernia. These frequent pathologies have to be taken into account when treating MP patients and in doubt, additional diagnostics like spinal imaging or X-rays of the hip and knee should be considered before surgical treatment, to ensure the diagnosis (Scholz et al., 2023). Stated preoperatively interventions raising patient satisfaction are a positive infiltration of the LFCN and an electromyography (EMG) to rule out other pathologies (Finerty et al., 2025). All patients within our cohort received an infiltration of the LFCN prior to surgery, whereas EMG was not usually applied. This could be an additional factor to raise patient satisfaction, whereas the reported frequency of EMG examinations within the review of Finerty et al. was only 14% of the cases (Finerty et al., 2025).

Special attention has to be given to traumatic or iatrogenic lesions, occurring e.g., after inguinal hernia surgery, iliac bone graft harvesting or hip arthroplasty. Those lesions are reasonable for up to 30% of all MP cases and the nerve can be already irreversibly damaged, so that the treatment plan has to be adapted with an overall higher risk for treatment failures (Benezis et al., 2007; de Ruiter et al., 2023).

Up to now, no consistent independent risk factors for treatment failure could be detected. Several factors like age, gender, duration of symptoms prior to surgery or obesity were evaluated as predictors for surgical outcome, whereas only obesity was significantly associated with a worse pain relief within one study (Siu and Chandran, 2005). Other studies found no significant correlation (Nouraei et al., 2007; Son et al., 2012). We found, that nicotine abuse was a risk factor for an unsatisfactorily outcome with an OR of 6.490 (p = 0.027). This might be induced by molecular dysregulations, especially affecting inflammation whilst axonal regeneration (Fang et al., 2023; Kim et al., 2018; Rodriguez-Fontan et al., 2020). Another statistically trend associated to an unsatisfactorily outcome was an increased duration of surgery (OR 4.902; p = 0.085), possibly due to atypical anatomical courses of the LFCN and therefore incomplete neurolysis. But, the results of our regression analysis should be interpreted with caution, because of the large confidence intervals and therefore restricted statistical strength. At least, the exact surgical technique may be an important factor for the outcome of the patients. Neurectomy is usually done through a transection of the nerve proximal of the inguinal ligament, but different techniques for the neurolysis are existing, which might lead to different treatment results (Malessy et al., 2019).

Another important outcome factor is the sensory function at the ventrolateral thigh. There is a broad discussion concerning the possibility to preserve sensation through neurolysis against a sensory loss in neurectomy, which might not bother the patient to any significant degree (Williams and Trzil, 1991). The neurolysis has the advantage to preserve or restore sensory function of the ventrolateral thigh (Reuter et al., 2025; Schwaiger et al., 2018). Reuter et al. showed 61.2% of patients after neurolysis with a complete sensory preservation (Reuter et al., 2025). In our patient cohort, 88 patients (81.5%) showed already hypoesthesia prior to surgery. Postoperatively, in 28 patients the hypesthesia disappeared, whereas eight patients showed a new sensory loss at the thigh (Fig. 4). 27 (43.5%) out of the 62 patients with a postoperative sensory disturbance found this bothersome, representing a relevant number of patients. All these patients were treated by neurolysis, no patient who suffered from the sensory loss received a neurectomy or neuroma resection. Because of the non-destructive concept of neurolysis, an increase of sensibility at the thigh over time might be still possible. We want to figure out, that the sensation at the thigh seems to be still a relevant outcome factor for the patients.

It is also worth mentioning, that the risk profile for both surgical interventions is very low. The most common reported complications are hematomas requiring nerve decompression surgery in 4.8 - 6.7%, marked subcutaneous effusions in 4.4%, wound healing disorders (2.4%) and wound infections (2.2%) (Rascón-Ramírez et al., 2022; Xu et al., 2021). We had only seven patients (6.5%) showing a wound healing disturbance, all treated by antibiotics. There was no patient who needed surgical revision. Within the literature and at least in our evaluation too, no patient experienced worsening of pain. Eight patients showed a new hypesthesia after the intervention, whereas only two of them found this bothersome.

Focusing on the health-related QoL, the SF-8 showed postoperatively in average similar measures as described for the normal population. The physical domain (PCS 45.64) was slightly below the mean for age-matched females (PCS 47.42) and males (PCS 48.28) within the normal German population, whereas the mental domain (MCS 52.49) was slightly above the normal population (females MCS 51.00; males MCS 51.86) (Ellert et al., 2005). Fig. 6 shows that the health-related QoL increases with the patient satisfaction after surgery, reflecting an efficient symptom reduction. EQ-VAS ratings and EQ index were also correlated to the patients’ satisfaction, which is not very surprising. It was remarkable, that satisfied patients had even higher EQ-VAS values (82.5 for PSI 1 and 80.0 for PSI 2) than the normal age-matched population with a mean of 72.9 (Buchholz and Janssen, 2023; Janssen and Szende, 2014). Unfortunately, only two third of the patients had available data for EQ-5D-5L and we could not receive data prior to surgery to compare for the effect of the operation. The EQ-5D-5L questionnaire was administered at only one of the two participating centers, so these results should be interpreted with caution. But we can state, that surgically treated MP patients can reach a health-related QoL similar to the normal population.

Our study has still some limitations beside the retrospective design with its selection bias as well as missing follow-ups and incomplete datasets. Subjective reported treatment outcomes, that are assessed some time after the procedure, may be skewed by the patient's perception, especially by other medical conditions that may have developed in the meantime or by changes in their life circumstances. We have to state a heterogeneity of the follow-up time due to the retrospective survey in 2024 and 2025. Therefore, we report patients with a follow-up of at maximum nearly 14 years that were treated in 2010 as well as patients with only a short follow-up of at minimum one month. We could not find any correlation of outcome and follow-up time. At least, the median 37.5 months can be stated as a middle-to long-term outcome. As a mentioned possible risk factor for treatment failure, we did not ask for the preoperative duration of symptoms, whereas this information in a retrospective manner, where the treatment took place years ago, usually reveals no reliable values. Additionally, we could not evaluate the course of the LFCN and possible atypical anatomical situations, that could influence the outcome. At least, no preoperative NRS values and QoL data were available to compare with the follow-up surveys.

As an outlook, to optimize the long-term course of surgically treated MP patients, subjective and objective outcome parameters have to be sampled in a prospective set-up and both common operative procedures have to be compared concerning their efficiency in pain response as well as for their side effects in a multicenter, randomized controlled fashion.

## 5 Conclusions

Surgical treatment of the lateral femoral cutaneous nerve in conservative therapy-refractory Meralgia paresthetica can significantly improve associated complaints in the majority of patients leading to a high number of satisfying results for these often chronically affected patients. Nicotine abuse seems to be a risk factor for an insufficient outcome.

## Declaration of competing interest

The authors declare that they have no known competing financial interests or personal relationships that could have appeared to influence the work reported in this paper.

## Appendix A Supplementary data

Supplement 1: Standardized documentation of the telephone survey with Patient Satisfaction Index (PSI). Additionally, the SF-8 and EQ-5D-5L were applied.
