# Supplementary material for: Symptom reduction and patient satisfaction after surgical therapy of Meralgia paresthetica – A bicentric retrospective analysis
Source: Brain Spine. 2026 Apr 18;6:106060. doi: 10.1016/j.bas.2026.106060 (PMC13141792; doi:10.1016/j.bas.2026.106060)
Supplement: Multimedia component 1 [file mmc1.docx]

1. **Are you satisfied with the surgery?**

| The surgery met my expectations. | □ |
| --- | --- |
| My symptoms did not improve as much as I had hoped after the surgery, but I would have the operation again for the same result. | □ |
| The surgery helped me, but I wouldn't have the surgery again for the same result. | □ |
| I have the same or worse symptoms than before the surgery. | □ |

1. **Did the surgery have any effect?**

| **No** | **Yes,**  **it was temporarily better for** | **Yes,**  **it was temporarily worse for** | **Yes, there was a sustained improvement of about** |
| --- | --- | --- | --- |
| □ | ____ months | ____ months | ____ % |

1. **How severe are your pain symptoms at present?** (0 = no pain / 10 = maximum)

|  | **0** | **1** | **2** | **3** | **4** | **5** | **6** | **7** | **8** | **9** | **10** |
| --- | --- | --- | --- | --- | --- | --- | --- | --- | --- | --- | --- |
| Pain at the groin at rest | □ | □ | □ | □ | □ | □ | □ | □ | □ | □ | □ |
| Pain at the groin under strain | □ | □ | □ | □ | □ | □ | □ | □ | □ | □ | □ |
|  |  |  |  |  |  |  |  |  |  |  |  |
| Pain at the thigh at rest | □ | □ | □ | □ | □ | □ | □ | □ | □ | □ | □ |
| Pain at the thigh under strain | □ | □ | □ | □ | □ | □ | □ | □ | □ | □ | □ |
|  |  |  |  |  |  |  |  |  |  |  |  |
| Which painkillers are you taking? |  | | | | | | | | | | |

1. **How is your sensory function of the ventrolateral thigh?**

| **Normal** | **Hypesthesia / tingling** | **Anesthesia** | **Does this disturbance bother you?** |
| --- | --- | --- | --- |
| □ | □ | □ | □ **Yes**  □ **No** |

1. **Did you have a wound healing disorder?**

| **Wound healing disorder** | □ **Yes** | □ **No** |
| --- | --- | --- |

1. **Do you also have complaints on the other side?**

| **Other side** | □ **Yes** | □ **No** |
| --- | --- | --- |

1. **How has your weight changed since the surgery?**

| **Weight change** | □ **less** | □ **more** | ____ ± kg |
| --- | --- | --- | --- |
